# 6-BA Delays the Senescence of Postharvest Cabbage Leaves by Inhibiting Respiratory Metabolism

**DOI:** 10.3390/foods13111607

**Published:** 2024-05-22

**Authors:** Cimei Wang, Yingying Yang, Jieting Yu, Zongli Liu, Wei Wei, Jianye Chen, Jianhua Zhu, Riming Huang

**Affiliations:** 1 Guangdong Provincial Key Laboratory of Food Quality and Safety, College of Food Science, South China Agricultural University, Guangzhou 510642, China; 13650707286@163.com (C.W.); 20233185078@stu.scau.edu.cn (J.Y.); 2 Guangdong Provincial Key Laboratory of Utilization and Conservation of Food and Medicinal Resources in Northern Region, School of Food Sciences and Engineering, Shaoguan University, Shaoguan 512005, China; 3 State Key Laboratory for Conservation and Utilization of Subtropical Agro-Bioresources, Guangdong Provincial Key Laboratory of Postharvest Science of Fruits and Vegetables, Engineering Research Center of Southern Horticultural Products Preservation, Ministry of Education, College of Horticulture, South China Agricultural University, Guangzhou 510642, China; yangyy1992@scau.edu.cn (Y.Y.); liuzongli@scau.edu.cn (Z.L.); weiwei@scau.edu.cn (W.W.); chenjianye@scau.edu.cn (J.C.)

**Keywords:** Chinese flowering cabbage, 6-BA, leaf senescence, activity

## Abstract

6-BA, a small molecule compound of cytokinins, has been proven to delay leaf senescence in different species, including Chinese flowering cabbage; however, its specific mechanism remains relatively unknown. In this study, the application of external 6-BA delayed leaf senescence in Chinese flowering cabbage, showing that 6-BA effectively prevented the decrease in the maximum quantum yield (Fv/Fm) and overall chlorophyll content and suppressed the expression of the senescence-associated gene *BrSAG12* over a 7-day period of storage. Moreover, treatment with 6-BA decreased the respiratory rate, NAD(H) content, the activities of hexose phosphate isomerase (PHI), succinate dehydrogenase (SDH), cytochrome c oxidase (CCO), and ascorbic acid oxidase (AAO) using enzyme-linked immunosorbent assay, and the transcriptional abundance of related genes by real-time quantitative polymerase chain reaction. Furthermore, 6-BA also increased the activity and expression levels of glucose-6-phosphate dehydrogenase (G6PDH) and 6-phosphate gluconate dehydrogenase (6-PGDH). The group treated with 6-BA retained elevated levels of NADP (H), ATP, total ATPase, and nicotinamide adenine dinucleotide kinase (NADK) activity, as well as the expression of respiratory enzymes. Molecular docking indicated that 6-BA hinders the glycolysis pathway (EMP), tricarboxylic acid cycle (TCA), and cytochrome pathway (CCP), and sustains elevated levels of the pentose phosphate pathway (PPP) through interactions with the PHI, SDH, 6-PGDH, G6PDH, CCO, and AAO proteins, consequently delaying postharvest leaf senescence in Chinese flowering cabbage.

## 1. Introduction

Vegetables are a main source of vitamins and nutrients, such as protein, minerals and phenols [1], and are essential in people’s daily lives. Chinese flowering cabbage (*Brassica rapa* ssp. *parachinensis*) is one of the most popular leafy vegetables and has important market value and economic value [2]. However, Chinese flowering cabbage respiration is vigorous, and its metabolism remains active after harvest. The rapid yellowing and senescence of leaves leads to the rapid loss of nutrients and flavour, resulting in considerable economic losses, which represents the main problem of vegetables after harvest and seriously restricts the healthy development of the vegetable industry [3,4]. Leaf senescence is a highly complex and programmed biological development process mediated by both internal and environmental factors, including the hydrolysis of pigments and proteins and the redistribution of nutrients [5]. Age, plant hormones, abiotic and biological stresses, energy, and various other factors regulate this process [6]. However, additional possible factors contributing to the deterioration of leaves after harvest have not been thoroughly explored. Therefore, thorough research on postharvest vegetable leaf senescence and its regulatory mechanism to reduce production and economic losses and promote the development of the vegetable industry is highly important.

Respiration and energy metabolism are important factors affecting the ageing and quality of postharvest fruits and vegetables. Moreover, plant respiration provides energy and intermediates for normal life activities and various physicochemical reactions of postharvest products. Changes in the plant energy state will also affect the intensity of respiratory metabolism, and the two affect each other [7,8].

Postharvest horticultural crops heavily rely on a respiratory metabolism, which leads to significant changes in intermediate metabolites across different pathways for metabolic and energy production [9]. The ageing process of harvested horticultural crops is connected to how quickly they respire and the ratio of various respiration pathways [10]. Common plant respiratory processes include the pentose phosphate pathway (PPP), tricarboxylic acid cycle (TCA), Embden–Meyerhof–Parnas pathway (EMP), and cytochrome pathway (CCP). PHI, SDH, G6PDH, and 6-PGDH, along with CCO and AAO, catalyse this process [9,11]. Previous studies have shown that higher respiratory rates, higher percentages of EMP and CCP, and lower percentages of PPP accelerate the senescence of postharvest horticultural crops such as tomatoes [12], Chinese flowering cabbage [4] and white mushrooms [10]. In addition, respiratory metabolic pathways are regulated by changes in respiratory metabolic enzyme activity, electron donors, and pyridine nucleotide content [9,13]. Furthermore, nicotinamide adenine dinucleotide (NAD(H)) and nicotinamide adenine dinucleotide phosphate (NADP(H)) play crucial roles as cytosolic electron donors in controlling plant respiratory metabolism and electron transport. NAD(H) plays a key role in the EMP and TCA respiratory pathways, whereas NADP(H) is primarily involved in the HMP respiratory pathway [8,9,14]. Changes in the environment, both inside and outside, can impact the activity of respiratory metabolism enzymes and the levels of NAD (H) and NADP (H), as well as the metabolic pathways of breathing. These alterations in respiration intensity subsequently influence the overall quality of harvested fruits and vegetables [7,9,15]. For example, melatonin treatment inhibited postharvest ageing of broccoli partly because melatonin significantly inhibited the activities of PHI, SDH and CCO and the content of NAD(H) in key enzymes of the EMP, TCA and CCP pathways. G6PDH and 6-PGDH enzymes were activated, leading to an increase in NADP(H) levels in the pentose phosphate pathway (HMP), as previously reported [16]. In contrast, hydrogen peroxide (H_2_O_2_) enhanced the respiratory metabolism and total respiratory intensity, increased substrate consumption and accelerated peel browning in postharvest litchi by inducing the EMP, TCA and CCP respiration pathways [9]. In addition to the influence of respiration intensity, fruit senescence and quality are also influenced by the cell energy state [17]. ATP becomes a common intermediate transmitter for the transfer of phosphate groups between upper- and down-stream high-energy phosphate compounds, which is catalysed by enzymes such as synthesis (ATP synthase) and dissipation (alternative oxidase, AOX) [4,18]. ATP carrier protein (AAC), a transmembrane protein on the mitochondrial membrane of eukaryotic organisms, mediates ADP/ATP exchange between mitochondria and the cytoplasmic matrix [19]. When fruits and vegetables enter senescence after harvest, the ATP content of cells decrease rapidly, such as in the case of Chinese flowering cabbage [4] and longan [20].

6-benzylaminopurine (6-BA) is a small cytokinin analogue. This method has been extensively used to slow the ageing process of different types of horticultural crops, such as Chinese chives [21] and Chinese flowering cabbage [22]. Past research has indicated that the role of 6-BA in plant ageing primarily centres on controlling the levels of senescence-related genes (*SAGs*) and the functions of enzymes that breakdown chlorophyll and remove free radicals [23,24]. In our previous study, transcriptome analysis revealed that 6-BA may delay leaf senescence in Chinese flowering cabbage after harvest by regulating chlorophyll metabolism, hormone metabolism, reactive oxygen species (ROS) homeostasis and respiratory metabolism. As individuals aged, there was an increase in the activity of genes encoding important enzymes involved in the respiratory process. However, the administration of 6-BA notably increased the expression of these genes [22]. Previous studies on the effect of 6-BA on delaying senescence and maintaining the storage quality of horticultural crops have focused on the regulation of antioxidant enzyme activities; while other pathways and molecular mechanisms of 6-BA on plant leaf senescence have been investigated, especially in leafy vegetables such as Chinese flowering cabbage, there is still a lack of systematic in-depth elucidation. However, it remains unclear whether 6-BA is involved in the respiration and energy status of postharvest flowering cabbage, which in turn delays leaf senescence. In addition, whether 6-BA, a small molecule compound, interacts with respiratory metabolic enzymes to change enzyme activity is also unknown. The aim of this study was to examine how exogenous 6-BA impacts respiration, metabolism, energy levels, enzyme activities, and gene expression in Chinese cabbage during postharvest storage, as well as the underlying regulatory processes involved. The findings of this research offer a fresh outlook on how 6-BA delays the ageing of plant leaves and establishes a new theoretical foundation for the impact of plant hormones on postharvest preservation. This is crucial for enhancing storage techniques, minimizing losses, and advancing the Chinese flowering cabbage industry.

## 2. Materials and Methods

### 2.1. Plant Materials and Treatments

Chinese flowering cabbage, scientifically known as *Brassica rapa* ssp., is a type of leafy green vegetable parachinensis. In this study, plants were purchased from a farm close to Guangzhou, China. Cabbages that were free of flowers, mechanical damage and rot were chosen and divided into two groups at random—one serving as the control group and the other serving as the treatment group. Then, each group (treatment group and control group) was divided into 3 batches (rebatch). Two groups of samples were soaked in 50 µM 6-BA and distilled water for 3 min, packed in baskets, sealed in polyethylene film bags, and stored in an incubator at 15 °C [22]. On Days 0, 1, 3, 5, and 7 of storage, the lower third leaf was collected for physiological examination and stored as frozen samples at −80 °C.

### 2.2. Total Chlorophyll Amount and Fv/Fm Relationships

The samples were extracted in 80% acetone and incubated overnight in the dark, after which the OD values at 663 and 645 nm were measured with a spectrophotometer [22]. The result of chlorophyll content was expressed as mg g^−1^ on a fresh weight basis. A chlorophyll fluorometer (Imaging-PAM-Maxi, Heinz Walz GmbH, Rohrdorf, Germany) was utilized for chlorophyll fluorescence imaging to capture and document the Fv/Fm ratio of variable fluorescence to maximum fluorescence [25].

### 2.3. Determination of the Respiration Rate

The breathing rate of the cabbage was indicated by the rate at which it released CO_2_. Three replicates were included in each treatment, with five cabbages in each replicate. The cabbages were placed in a sealed tank for 3 h. Next, 1 millilitre of gas was extracted and analysed using a GC-17A gas chromatograph (Shimadzu Corporation, Kyoto, Japan)**.** The unit of results was mg CO_2_ kg^−1^ FW h^−1^ [4].

### 2.4. Assessment of Enzyme Activity Associated with Respiratory Metabolism

Kits from Suzhou Keming Biotechnology Co., Ltd. in Suzhou, China, were used to identify PHI, SDH, CCO, and AAO activities. The G6PDH activity test kit 6-PGDH was purchased from Grace Biotechnology Co., Ltd. (Suzhou, China) and all specific tests were conducted following the provided instructions. Then, the enzyme activities were calculated. The measurement was denoted as U per milligram of protein.

### 2.5. Measurement of NADK Enzyme Activity and the Levels of NAD(H) and NADP(H)

The NADK activity and the NAD^+^, NADH, NADP^+^ and NADPH contents were determined using a biochemical kit produced by Grace Biotechnology Co., Ltd. The procedures were performed in accordance with the provided guidelines. NADK activity was quantified as the production of NADP^+^ at a rate of 1 nmol per gram of protein per minute, with the measurement reported as U g^−1^ protein. NAD content was the sum of NAD^+^ and NADH, NADP was the sum of NADP^+^ and NADPH, and the contents of NAD(H) and NADP(H) were expressed as μmol kg^−1^ FW.

### 2.6. Determination of Total ATPase Activity and ATP Content

The total amount of ATP was determined using a biochemical kit produced by Grace Biotechnology Co., Ltd. The results are expressed as U per milligram of protein. ATP total enzyme activity was measured using a biochemical kit from Nanjing Jiancheng Bioengineering Co., Ltd. in Nanjing, China. The principle was that ATPase can decompose ATP to generate ADP and inorganic phosphorus. The ATPase activity can be assessed by quantifying the level of inorganic phosphorus. The outcome was reported in milligrams per kilogram of fresh weight.

### 2.7. Real-Time Quantitative Polymerase Chain Reaction (qPCR)

RT–qPCR was carried out as previously described by Fan et al. [24]. Gene expression levels were determined by employing the 2^−ΔΔCt^ relative method for calculation. All gene-specific primers used are listed in Table S1.

### 2.8. Molecular Docking

AlphaFold (AlphaFold.ebi.ac.uk, accessed on 16 to 18 April 2023), which is based on deep learning, was used to model cabbage-related proteins to obtain more accurate three-dimensional protein structures [26]. A combination of homology modelling (https://swissmodel.ExPASy.org/interactive, accessed on 19 to 22 April 2023) and nonhomologous modelling methods (https://yanglab.qd.sdu.edu.cn/trRosetta/help/index.html, accessed on 19 to 22 April 2023) were used to predict the structures of proteins with unclear crystal structures [27,28].

6-Benzylaminopurine was used as a model ligand and different proteins were used as receptors. To evaluate the binding energy and interaction mode between the ligand and receptor, the silico protein–ligand docking software Autodock Vina 1.2.2 (https://www.dockeasy.cn/, accessed on 23 April 2023), which is used for molecular docking, was used to assess the binding energy and interaction mode of the ligand and receptor in silico [29]. The grid box dimensions were 30 angstroms by 30 angstroms by 30 angstroms, with a grid point spacing of 0.05 nanometres.

The Protein–Ligand Interaction Profiler was utilized to detect noncovalent interactions between biological macromolecules and their ligands, as well as to examine the crucial amino acids participating in ligand–receptor interactions. Affinities of less than or equal to −4.25 kcal/mol, −5.0 kcal/mol, and −7.0 kcal/mol suggest potential binding, favourable binding, and robust binding affinity between ligands and receptors, respectively.

### 2.9. Statistical Analysis

Analysis was conducted using Student’s *t* test or one-way ANOVA in SPSS software (IBM SPSS Statistics 20). Significant differences were identified based on the *p* value (*p* < 0.05 or *p* < 0.01).

## 3. Results

### 3.1. The Impact of External 6-BA Application on Postharvest Cabbage Leaf Senescence

**Figure 1:**
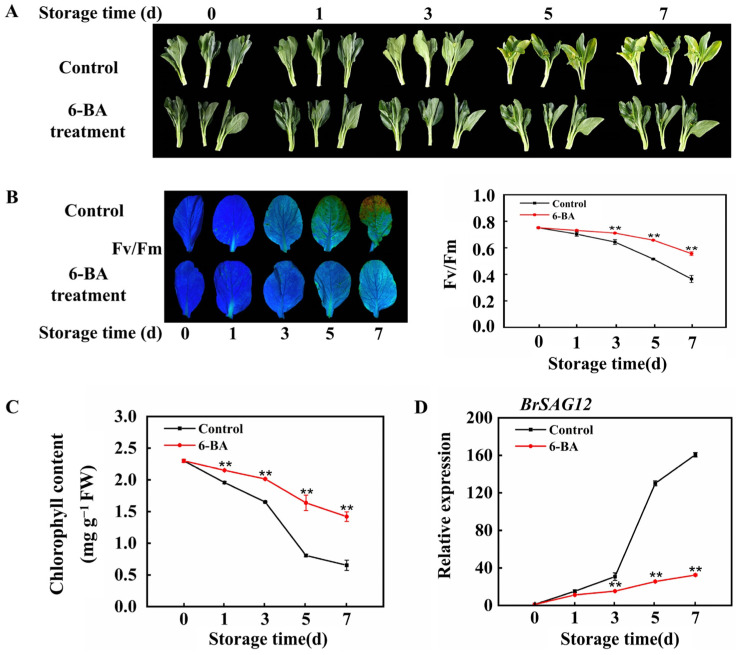
The use of 6-BA prevents the breakdown of chlorophyll and slows the senescence process of postharvest Chinese flowering cabbage leaves. (**A**) Alterations in the visual characteristics of control and cabbage leaves treated with 6-BA as they aged; (**B**) chlorophyll fluorescence imaging was conducted on cabbage leaves treated with 6-BA and control leaves to observe changes in Fv/Fm; (**C**) changes in chlorophyll content; (**D**) transcriptional level of *SAG12*. The data represent the average plus or minus standard error of three biological replicates. Statistically significant differences between treatments are denoted by asterisks, with ** representing *p* < 0.01.

Figure 1A indicated that the leaves in the control group started to show noticeable yellowing by the third day of storage, whereas the leaves in the treatment group began to exhibit significant yellowing by the fifth day. With increasing storage time, the degree of leaf yellowing in both groups gradually increased. By the seventh day of storage, all the leaves in the control group had turned a bright yellow colour. Fluorescence images varied in colour from blue to green and brown, showing the breakdown of chlorophyll; this reflected a decrease in chlorophyll content and a rapid decrease in the Fv/Fm ratio of the control leaves during storage (Figure 1B). With the change in phenotype, a large amount of chlorophyll in the control group dramatically decreased on the fifth day. The chlorophyll levels in the untreated group decreased from 2.30 mg g^−1^ at harvest to 0.81 mg g^−1^ by the fifth day, whereas the chlorophyll levels in leaves treated with 6-BA decreased by only 29% (Figure 1C). Furthermore, there was a notable increase in the expression of the senescence marker gene (*BrSAG12*) throughout the storage period. After the application of 6-BA, the increase in *BrSAG12* expression was significantly inhibited (Figure 1D).

### 3.2. The Impact of External 6-BA Application on Respiratory Metabolism

**Figure 2:**
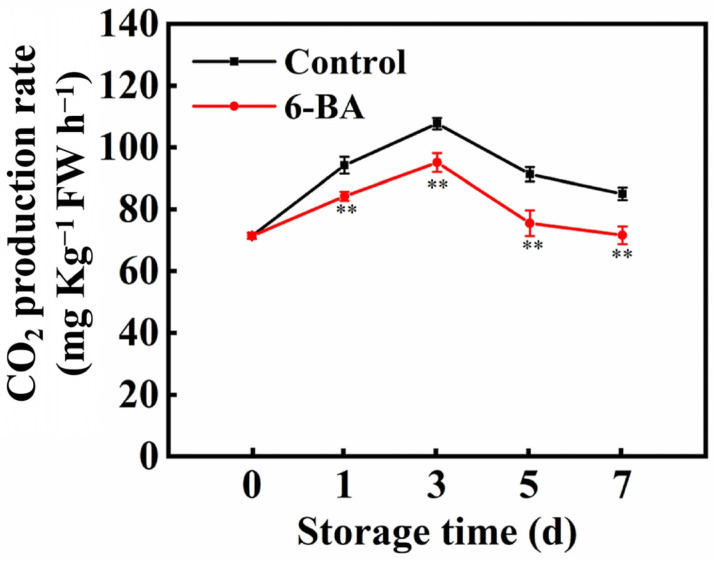
Impact of external application of 6-BA on the rate of respiration in Chinese flowering cabbage after harvest. The data represent the average plus or minus standard error of three biological replicates. Statistically significant differences between treatments are denoted by asterisks, with ** representing *p* < 0.01.

Figure 2 showed that the respiratory activity of cabbage after harvest followed a pattern of initial growth followed by a decrease over time during storage. The respiratory intensity of the control leaves was greatest on the third day of storage and then decreased. Respiratory activity was lower in the 6-BA treatment group than in the control group over the first 1–7 days of storage. By Day 7, the respiration rate of the untreated leaves was 16% greater than that of the leaves treated with 6-BA.

**Figure 3:**
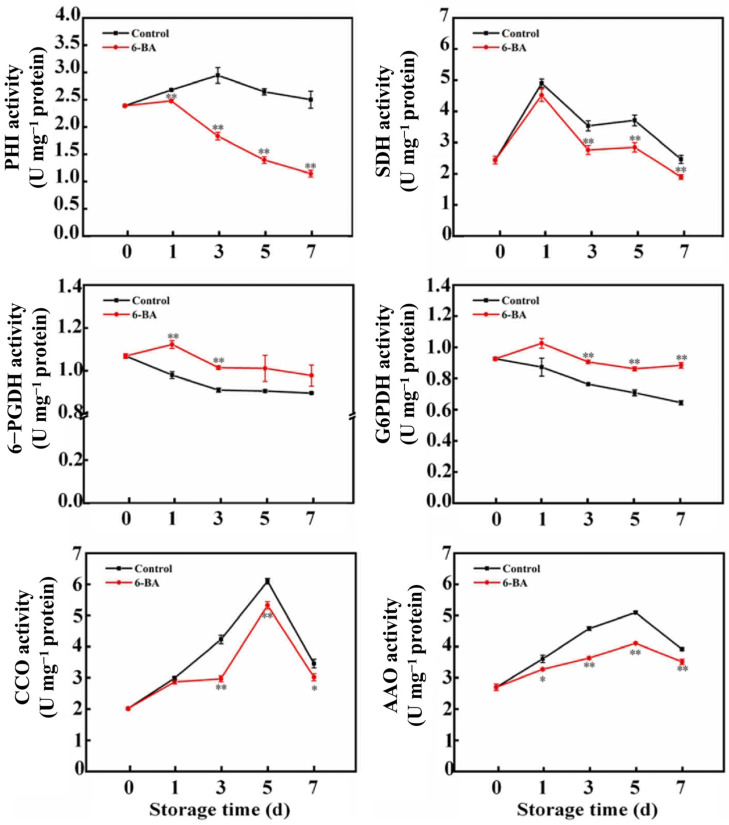
The impact of external 6-BA on the activities of enzymes related to respiration, such as PHI, SDH, 6-PGDH, G6PDH, CCO, and AAO, in Chinese flowering cabbage. The data represent the average plus or minus standard error of three biological replicates. Statistically significant differences between treatments are denoted by asterisks, with * representing *p* < 0.05 and ** representing *p* < 0.01.

Figure 3 displayed the findings from determining the activity of crucial enzymes in the respiratory pathway. The enzyme PHI, a crucial component of the EMP pathway according to Lin et al. [9], exhibited an initial increase in activity followed by a decrease as storage time increased in cabbage leaves treated with both 6-BA and the control. On Day 7, the PHI activity of the treated leaves decreased by 54.2% compared with that of the control leaves. According to Liu et al. [30], the activity of succinate dehydrogenase, a crucial enzyme in the tricarboxylic acid cycle, exhibited a pattern of initial growth followed by a decrease. SDH levels increased on the first day of storage, followed by a gradual decrease over time. By the fifth and seventh days, SDH activity in the treatment group had decreased by 23.3% and 23.0%, respectively, compared to that in the control group. 6-Phosphogluconate dehydrogenase and glucose-6-phosphate dehydrogenase play crucial roles in the pentose phosphate pathway, as highlighted in studies by Guo et al. [12] and Lin et al. [9]. The activity of 6-PGDH generally decreased slowly at the initial stage of storage. The activity of the control group decreased at the beginning of storage before levelling off. On Day 1 and Day 3, compared with those in the control group, the 6-PGDH activity in the treatment group increased by 14.5% and 11.5%, respectively. The activity of G6PDH was elevated by the 6-BA treatment, surpassing that of the control throughout the entire storage duration. Changes in the activities of CCO and AAO, important respiratory terminal enzymes, are also important factors affecting respiratory metabolism [9]. CCO levels initially increased before decreasing over the entire duration of storage. By the seventh day of storage, there was a decrease in CCO activity, with the 6-BA treatment group still showing a 12.8% reduction compared to the control group. In all treatment groups, the AAO activity was consistently lower than that in the control group. AAO activity decreased by 19.4% and 10.3% on Days 5 and 7, respectively. The above results indicated that 6-BA treatment also inhibited the activity of CCO and AAO during the senescence of cabbage leaves.

### 3.3. Impact of 6-BA on the Transcription Levels of Enzymes Involved in Respiratory Metabolism

**Figure 4:**
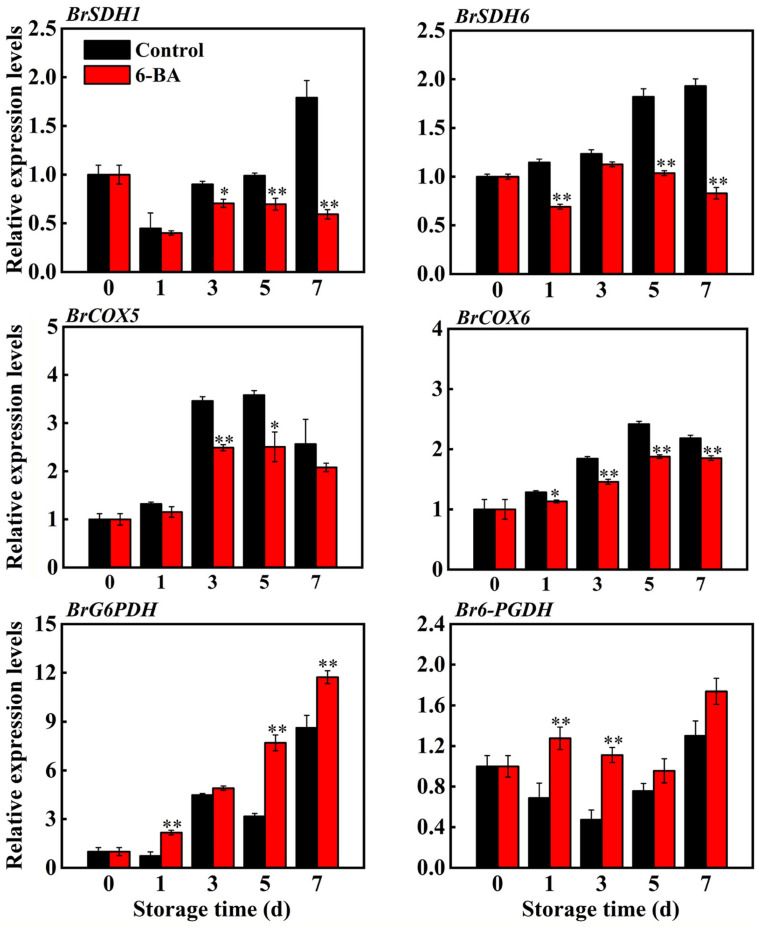
The impact of external 6-BA on the transcription of genes that encode enzymes related to respiration, such as *BrSDH1*, *BrSDH6*, *BrCOX5*, *BrCOX6*, *BrG6PDH* and *Br6-PGDH*. The data represent the average plus or minus standard error of three biological replicates. Statistically significant differences between treatments are denoted by asterisks, with * representing *p* < 0.05 and ** representing *p* < 0.01.

Additional analysis of the gene expression of the SDH, CCO, 6-PGDH, and G6PDH enzymes (*BrSDH1*, *BrSDH6*, *BrCOX5*, *BrCOX6*, *Br6-PGDH*, and *BrG6PDH*) was conducted. Figure 4 shows that the use of 6-BA suppressed the activity of *BrSDH1*, *BrSDH6*, *BrCOX5*, and *BrCOX6*, resulting in reductions of 29.6%, 43.1%, 30%, and 22.4%, respectively, compared to those of the control group after 5 days. In leaves treated with 6-BA, the levels of *BrG6PDH* and *Br6-PGDH* were elevated compared to those in the control.

### 3.4. The Impact of External 6-BA Application on NADK Activity and the Levels of NAD(H) and NADP(H)

**Figure 5:**
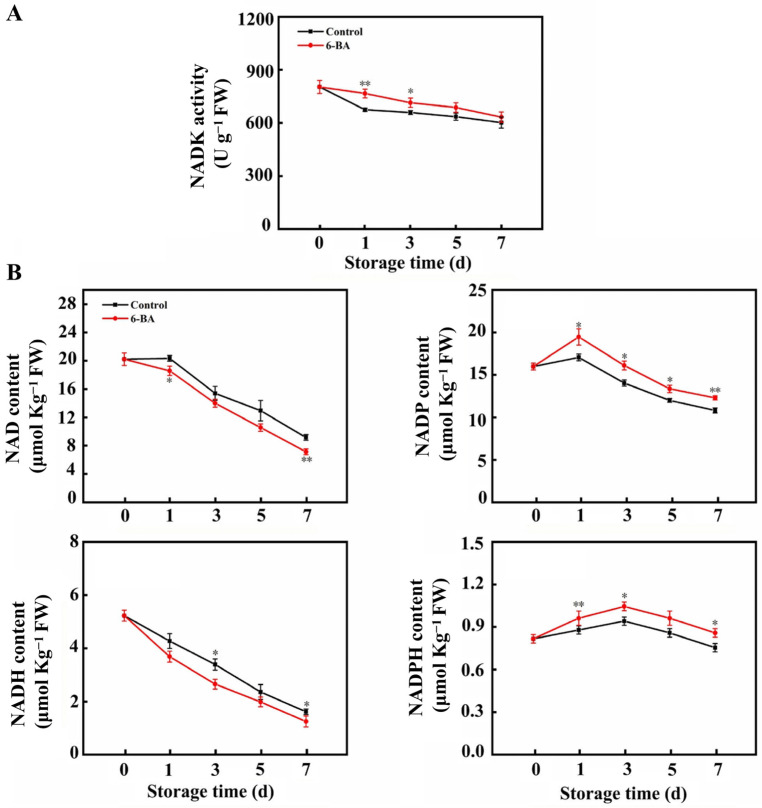
The impact of external 6-BA on the activity of NADK (**A**) and the levels of NAD, NADH, NADP, and NADPH (**B**) in Chinese flowering cabbage. The data represent the average plus or minus standard error of three biological replicates. Statistically significant differences between treatments are denoted by asterisks, with * representing *p* < 0.05 and ** representing *p* < 0.01.

Figure 5A illustrated that NADK activity decreased over time in the control samples but remained stable and did not decrease with 6-BA treatment. On the initial day, the NADK enzyme activity in leaves treated with 6-BA was 13.6% greater than that in the control group. As shown in Figure 5B, the contents of NAD (H) and NADP (H) decreased gradually with increasing storage time. By the seventh day, the NAD and NADH levels in the experimental group were reduced by 22% and 22.7%, respectively, compared to those in the control group. In the treatment group, there was a 13.9% increase in NADP levels and a 13.8% increase in NADPH levels. Cabbage leaves treated with 6-BA had lower levels of NAD and NADH than those in the control but maintained higher levels of NADP and NADPH, possibly because of the increased NADK activity induced by 6-BA.

### 3.5. The Impact of External Application of 6-BA on Total ATPase Activity, ATP Levels, and the Regulation of Genes Related to Energy Metabolism in Postharvest Cabbage

**Figure 6:**
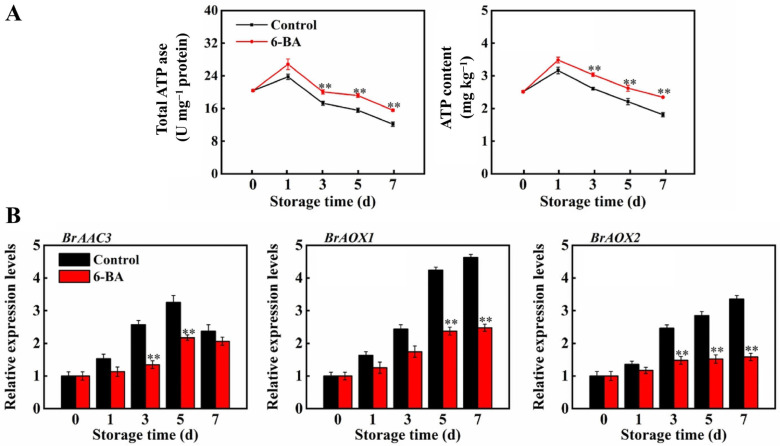
Impact of external 6-BA on overall ATPase function and ATP levels in Chinese flowering cabbage (**A**). The impact of external 6-BA on the expression of genes encoding ATP-related enzymes, such as *BrAAC3*, *BrAOX1*, and *BrAOX2* (**B**). The data represent the average plus or minus standard error of three biological replicates. Statistically significant differences between treatments are denoted by asterisks, with ** representing *p* < 0.01.

During storage, we observed alterations in the overall ATPase activity and ATP levels in leaves, and the findings are displayed in Figure 6A. The activity of the total ATPase enzyme and the ATP content first increased and then decreased. On Days 3, 5, and 7, the overall ATPase activity increased by 16%, 23.2%, and 28.3%, respectively, compared to that of the control. The change in ATP content showed the same trend. The ATP content in the control leaves continued to decrease after 1 day of storage, while 6-BA treatment slowed the decrease in ATP. The ATP content was still 29.8% greater than that in the control on Day 7. As shown in Figure 6B, the transcription levels of *BrAAC3*, *BrAOX1* and *BrAOX2* were further detected. The expression of *BrAAC3* first increased and then decreased. It was inhibited after 6-BA treatment during storage. The expression of *BrAOX1* and *BrAOX2* increased during storage, and the expression levels increased throughout storage, peaking on the seventh day, with decreases of 46.5% and 52.8%, respectively, compared to those in the control.

### 3.6. Molecular Docking Analysis of the Interaction between 6-BA and Respiratory Metabolic Enzymes

**Figure 7:**
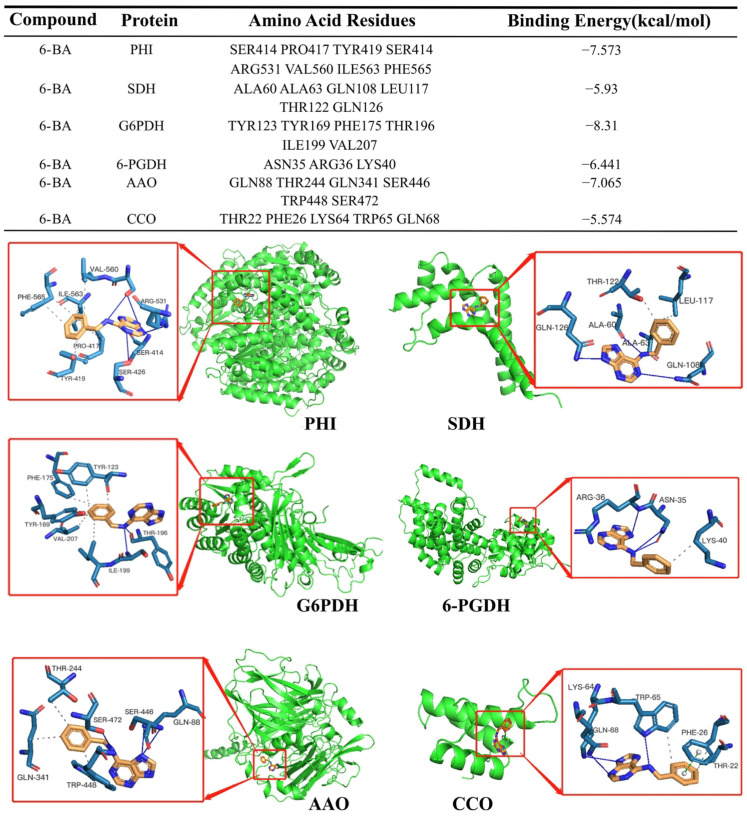
Schematic diagram of the molecular docking prediction of 6-BA and key proteins (PHI, SDH, G6PDH, 6-PGDH, AAO and CCO) in the respiratory pathway.

6-BA was docked with different cabbage proteins, and the docking results were shown in Figure 7. 6-BA forms two hydrogen bonds with TYR196 in G6PDH as well as with ILE199, and 6-BA binds to TYR123, TYR169, PHE175, ILE199, and VAL207 through hydrophobic interactions. Among them, 6-BA forms both hydrogen bonds and hydrophobic interactions with ILE199. 6-BA forms one hydrogen bond with SER414 and ARG531 in PHI and two hydrogen bonds with VAL560. 6-BA is bound by hydrophobic interactions with PRO417, PHE565, ILE563, and VAL560. Among them, 6-BA forms both hydrogen bonds and hydrophobic interactions with VAL560. 6-BA forms one hydrogen bond with GLN88, SER446, and SER472 in AAO. 6-BA is bound to THR244, GLN341, and TRP448 via hydrophobic interactions. 6-BA forms three hydrogen bonds with ASN35 of 6-PGDH, and 6-BA binds to LYS40 through hydrophobic interactions. 6-BA forms a hydrogen bond with ALA63, GLN108, and GLN126 of SDH, and 6-BA binds to THR122 and LEU117 through hydrophobic interactions. 6-BA forms one hydrogen bond with each of ALA 63, GLN108, and GLN126 in SDH, and 6-BA binds to THR122 and LEU117 through hydrophobic interactions. 6-BA forms one hydrogen bond with TRP65 of CCO and two hydrogen bonds with GLN68. 6-BA is bound to TRP65 and PHE26 by hydrophobic interactions. 6-BA interacts with TRP65 through both hydrogen bonding and hydrophobic interactions, among others.

Further analysis of the molecular docking results showed that 6-BA had strong binding activity with G6PDH, PHI and AAO, which proved that G6PDH, PHI and AAO were the main enzymes affecting the metabolic pathway of cell respiration to a certain extent. 6-BA has good binding activity with 6-PGDH, SDH and CCO, and it also plays a certain role in affecting the metabolic pathway of cell respiration. This means that 6-BA may interact more strongly with G6PDH, the PHI, and AAO. However, molecular docking analysis only provided a theoretical basis, and the interaction between 6-BA and respiratory metabolic enzymes still needs to be verified experimentally.

## 4. Discussion

Previous studies have shown that the greater the respiration of fruits and vegetables during storage is, the faster the ageing and the shorter the storage life [15]. Therefore, in the storage process of fruits and vegetables, to ensure normal respiratory metabolism, storage resistance and disease resistance, all possible technical measures should be taken to reduce respiratory intensity and inhibit the ageing process. For example, the air-conditioned storage of broccoli [7] and white mushrooms [10] and nanocomplex-packaged *Flammulina oryzae* [15] can delay the ageing process by reducing the respiratory intensity and changes in respiratory metabolic pathways during storage to varying degrees.

The aim of this study was to investigate whether 6-BA can slow the ageing of cabbage by controlling respiratory metabolism. Initially, we examined the changes in the respiratory activity of cabbage in both the control and 6-BA treatment groups. The results showed that 6-BA treatment successfully suppressed the increase in respiratory activity as the cabbage leaves aged (Figure 2). H_2_O_2_ treatment enhanced the postharvest respiratory metabolism of litchi, mainly because H_2_O_2_ treatment increased the activities of the EMP, TCA and CCP respiratory metabolic pathways while inhibiting the activities of HMP respiratory pathways. Moreover, the postharvest respiration intensity of litchi was enhanced [9]. Consistent with the above results, 6-BA treatment decreased the respiratory intensity of postharvest cabbage leaves (Figure 2) and decreased the activities of the key respiratory pathway enzymes PHI, SDH, CCO and AAO (Figure 3) and the expression of *BrSDH1*, *BrSDH6*, *BrCOX5* and *BrCOX6* (Figure 4). The activity of G6PDG, 6-PGDH and NADK increased (Figure 3 and Figure 5), and the expression of *BrG6PDH* and *Br6-PGDH* increased (Figure 4); moreover, the content of NADP(H) increased, and that of NAD(H) decreased (Figure 3, Figure 4 and Figure 5). Therefore, the EMP, TCA and CCP respiratory pathways were reduced, the HMP respiratory pathway was enhanced, the respiratory metabolism was reduced, the substrate consumption was reduced, the product quality was maintained, and the senescence of cabbage leaves was delayed. In this study, we not only systematically investigated the physiological effects of 6-BA treatment on different respiratory metabolic pathways in cabbage but also revealed that 6-BA can reduce respiratory metabolism by regulating the activity of different respiratory pathways and reducing respiratory intensity during storage. It was also found that the influence of 6-BA on these respiratory pathways may be related to its regulation of the expression of key enzyme-encoding genes of the corresponding respiratory pathways. These results further deepen the understanding of the physiological and molecular mechanisms by which 6-BA affects respiratory metabolism during leaf ageing.

An insufficient energy supply or decreased energy production efficiency will not only accelerate respiratory metabolism but also trigger the destruction of cell membrane structural integrity, resulting in rapid ageing and a decrease in the quality of postharvest fruits and vegetables [31]. Therefore, energy deficit is considered to be one of the key factors leading to ageing and a reduction in the quality of fruits and vegetables [15]. In this study, with increasing storage time, the leaves of the cabbage gradually yellowed and aged, and the ATP content of the leaves continued to decrease (Figure 6A). The postharvest ageing of horticultural products such as postharvest longan [8] and broccoli [16] is also associated with decreased energy levels. We found that 6-BA treatment inhibited the decrease in ATP levels, which may be related to the maintenance of the increased total ATPase activity (Figure 6A). An increased ATPase activity is also beneficial for maintaining intracellular energy levels, ion homeostasis and mitochondrial integrity [32]. Exogenous 6-BA treatment improved the energy level of postharvest cabbage, and a sufficient energy level reduced the demand for energy generation by cells, thus reducing the feedback to respiratory metabolism. Based on these results, it can be inferred that 6-BA treatment results in lower respiratory metabolism, which is closely related to the maintenance of a higher energy state. In the study, the expression levels of *BrAOX1* and *BrAOX2* increased with leaf ageing during storage in postharvest Chinese cabbage, while 6-BA significantly inhibited the expression of these genes (Figure 6B). Therefore, 6-BA treatment delayed the senescence of postharvest cabbage leaves and maintained higher quality, which may be partly because 6-BA treatment maintained a higher energy state of postharvest cabbage leaves while reducing their respiratory metabolism and consumption. This finding suggested that the regulatory effect of 6-BA on respiration and energy metabolism may be important for delaying the ageing of fruits and vegetables.

Through further analysis of the molecular docking results, we found that the hydrogen bonding of 6-BA to respiratory metabolic enzymes mainly involves several key amino acid residues of these proteins. The crucial function of proteins is influenced by certain amino acids, and their interaction with 6-BA could impact activity levels, ultimately affecting respiratory signalling pathways and influencing the ageing process. This result provides early support for the subsequent concrete exploration.

## 5. Conclusions

**Figure 8:**
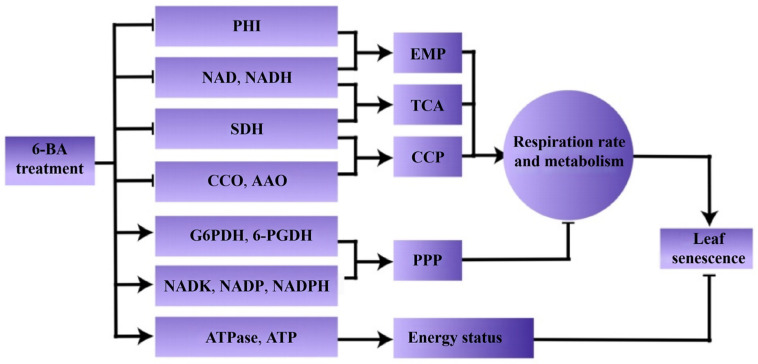
A model of 6-BA delayed the senescence of postharvest cabbage leaves by inhibiting respiratory metabolism.

In conclusion, this research demonstrated that applying external 6-BA helped to delay leaf ageing in Chinese flowering cabbage plants. Moreover, treatment with 6-BA suppressed the rate of respiration, the levels of NAD(H), and the activities of the enzymes PHI, SDH, CCO, and AAO, as well as the expression of related genes. Furthermore, 6-BA increased the activity and expression levels of G6PDH and 6-PGDH. The group treated with 6-BA retained elevated levels of NADP(H), ATP, total ATPase, and NADK activity, as well as the expression of respiratory enzymes. This work proposed a novel model (Figure 8), providing new insights of 6-BA delayed the senescence of postharvest cabbage leaves by inhibiting respiratory metabolism. Molecular docking indicated that 6-BA hinders the EMP, TCA, and CCP pathways and sustains elevated PPP levels through interactions with the PHI, SDH, 6-PGDH, G6PDH, CCO, and AAO proteins, consequently postponing postharvest leaf senescence in Chinese flowering cabbage.

## Data Availability

The original contributions presented in the study are included in the article/Supplementary Materials, further inquiries can be directed to the corresponding authors.

## Supplementary Materials

The following supporting information can be downloaded at: https://www.mdpi.com/article/10.3390/foods13111607/s1, Table S1: Summary of primers used in this study; Table S2: Genome IDs and description of 6 target proteins in this study; Text S1: The amino acid sequence of a respiratory metabolic enzyme for molecular docking.
