# Supplementary material for: 6-BA Delays the Senescence of Postharvest Cabbage Leaves by Inhibiting Respiratory Metabolism
Source: Foods. 2024 May 22;13(11):1607. doi: 10.3390/foods13111607 (PMC11171477; doi:10.3390/foods13111607)
Supplement: Supplementary file 1 [file foods-13-01607-s001.zip › foods-2962343-supplementary.pdf]

Table S1. Summary of primers used in this study.

Assay	Primer sequence (5'-3')	
qRT-PCR	*BrActin-qF: CGCTTAACCCGAAAGCTAAC*	*BrActin-qR: TACGCCCACTAGCGTAAAG*
	BrSAG12-qF:TTTCTTCTGGTGCTCTGCC	*BrSAG12-qR:CGCCCAACAACATCCACAACT*
	BrSDH1-qF:AAGTTTCGAGGATGTCCAG	*BrSDH1-qR:AAGCGTTTATCAATAGGTTC*
	BrSDH6-qF:GGTCGTTTGCGGATGGATT	*BrSDH6-qR:AGAAGGAAGAGGCGTGTCG*
	BrCOX5 -qF:TTTTGAATGCCCTGTTTGC	*BrCOX5-qR:GTGGTGGTCGTCTTCATCG*
	BrCOX6-qF:GCTTTAGCCGCCTATGTTT	*BrCOX6-qR:AGTGCCCTTCGTTGTGCTT*
	BrG6PDH-qF:AGGTCATAGCAAGGGTGGA	*BrG6PDH-qR:CCGCAGCAAATGTAGGAGT*
	Br6-PGDH-qF:AAGGGTGATGGGCATTTGG	*Br6-PGDH-qR:TGGTGGGAGACGGAACAGA*
	BrAAC3-qF:TAAGAAAGAAGGAGCCAAGT	*BrAAC3-qR:TTGTCATAGCCAGCGAGTA*
	BrAOX1-qF:GAAGTCGCTAAACCCAAAT	*BrAOX1-qR:ATCAAGTAACCGAGGAAAT*
	BrAOX2-qF:CACTGTGATTCGTGCTGAT	*BrAOX2-qR:TTCTCGCAACTCCTTCCCT*

Table S2. Genome IDs and description of 6 target proteins in this study.

Gene location	Protein	Description	Symble
BraA09g019380.3C	PHI	glucose-6-phosphate isomerase, cytosolic	PGIC
BraA06g005640.3C	SDH6	succinate dehydrogenase subunit 6, mitochondrial	SDH6
BraA08g009110.3C	G6PDH	glucose-6-phosphate 1-dehydrogenase 1, chloroplastic	G6PD1
BraA01g044410.3C	6-PGDH	6-phosphogluconate dehydrogenase, decarboxylating 2	PGD2
BraA01g036550.3C	CCO	cytochrome c oxidase subunit 5b-1, mitochondrial isoform X1	COX5B-1
BraA06g044860.3C	AAO	L-ascorbate oxidase homolog	AAO

Text S1. The amino acid sequence of a respiratory metabolic enzyme for molecular docking.

>BraA09g019380.3C, PHI

MASPTALISETQAWKDLKGHVEDINKTHLRDLMSDAHRCESMMLEFEGLLLDYSRQRATVETMDKLLNL
AKAAHLSEKISRMFNGEHINSTENRSVLHVALRAPKDAVIKADGKNVVPEVWNVLDKIKDFSEKIRSGSW
VGATGKPLKDVIAIGIGGSFLGPLFVHTALQTDPEAAECAKGRQLRFLANIDPVDVARNINGLNPETTLVVV
VSKTFTTAETMLNARTLRNWITTALGAPAVAKHMVAVSTNLALVEKFGIDPNNAFAFWDWVGGRYSVCS
AVGVLPLSLQYGFSVVEKFLKGASSIDQHFQSTPFEKNIPVLLGLLSVWNVSFLGYPARAILPYSQALEKFA
PHIQQVSMESNGKGVSIDGLPLPFETGEIDFGEPGTNGQHSFYQLIHQGRVIPCDFIGTVKSQQPVYLEGEV
VSNHDELMSNFFAQPDALAYGKTPEQLQKENVSENLIPHKTFSGNRPSLSLLLPELSAYNVGQLLAIYEHR
VAVQGFVWGINSFDQWGVELGKVLATQVRKQLHSSRTQGAAPEGFNYSTTTLLKRYLEVNIAFSVVFIGF
SSPYD*

>BraA06g005640.3C, SDH6

MGDSRSFADGFKGFWEERLSFLENYTRFTKRDTPLPSWSSSDVEEFIASDPVHGPTLKTAREAATFGVTGA
ALGALSTAAFAWRYSKSPHGAALSFLGGGVFGWTFGQEVANHTLQLYKLDTMAAQVKFMEWWERKTQ
*

>BraA08g009110.3C, G6PDH

MATHTMIVRSSSSALAASSPLKETLPLFTTRSLTFPRKSSFSRLRLRFFAEKLSQLDSSNGCASLQDSGEHLT
EEHDTKEESSTLSITVVGASGDLAKKKIFPALFALFYEGCLPQDFSVFGYARTKLTHEELRVMISRTLTCRID
QRENCVDKMDQFLKRCFYHSGQYNSEDDFAELNTKLNEKELSNRLYYLSIPPNIFVDVVRCASLRASSVN
GWTRVIVEKPFGRDSESSGELTRCLKQYLTEEQIFRIDHYLGKELVENLSVLRFSNLVFEPLWSRNYIRNVQ
LIFSEDFGTEGRGGYFDQYGIIRDIMQNHLLQILALFAMETPVSLDAEDIRSEKVKVLRSMKPLLLQDMIVG
QYKGHSKGGKAYPGYTDDPTVPTNSLTPTFAAAAMFINNARWDGVPFLMKAGKALHTRGAEIRVQFRHV
PGNLYKKNFATDLDKATNELVIRVQPDEGIYLRINNKVPGLGMRLDRSDLNLLYRSRYPREIPDAYERLLLD
AIEGERRLFIRSDELDAAWDLFTPALKELEHKKIVPELYPYGSRGPVGAHYLASKYNVRWGDLEDA*

>BraA01g044410.3C, 6-PGDH

MAVQPTRIGLAGLAVMGQNLALNIASKGFPISVYNRTTSKVDETVERAKKEGNLPVYGFHDPESFVNSIQK
PRVIIMLVKAGAPVDQTIKTLSAYLEKGDCIVDGGNEWYENTERREKAVAENGFLYLGMGVSGGEEGARN
GPSMMPGGSFEAYKNIEDILLKVAAQVRDSGPCVTYIGKGGSGNFVKMVHNGIEYGDMQLIAEAYDVLK
SVGKLTNEELHGVFTEWNKGELESFLVEITADIFGIKDDKGDGHLVDKVLDKTGMKGTGKWTVQQAAEL
SVPSPTIESSLDARFLSGLKDERVQAAKVFKEGGFGDVLTDQTVDKKQLIDDVRKALYASKICSYAQGMNL
IRAKSVEKGWGLKLGELARIWKGGCIIRAIFLDRIKQAYDRNAELANLLVDPEFAKEIIERQSAWRRVVCLS
INSGISTPGMSASLAYFDSYRRERLPANLVQAQRDYFGAHTYERTDMEGSFHTEWFKIARQSKM*

>BraA01g036550.3C, CCO

MWRRIVSSHLKSLAADVAAASPRRSIATTTARPVGFHLAANRSAVSASSFLTPRHFSSESVESVAKKKVED
VMPIATGHEKEELEAELEGRRLLDIDFPEGPFGTKESPAIVKSYYDKRIVGCPGGEGEDEHDVVWFWLEKG
KSFECPVCTQYFELEVVGPGGPPDGHGDEDDHH*

>BraA06g044860.3C, AAO

MGWWLKYCGLWTVMTIIVSLVQAEDPYRFFDWRVTYGNIYPLGIPQRGILINGQFPGPEIYSVTNDNLIIN
VHNDLDEPFLLSWNGVQLRKNSYQDGVYGTTCPIPPGKNYTYAIQVKDQIGSFFYFPSLAFHKAAGAFGG
LRVLSRPRIPVPFPEPAGDFTFLIGDWYSQHDHKNLKALLDRGHRLPFPDKVLINGNGVNFSSSLTVHKGK
TYRFRISNVGLQHSLNFKIVDHQMKLVEVEGTHTIQSMYSSLDIHVGQSYSVLVTMDQPEKDYSIVVATR
FAAKKILVGSTLHYSNSRQSLSSASLSARGPADELDWSIKQARSIRTNLTASGPRPNPQGSYHYGLINISRT
LVLESSAGLVKRKQRYAINGVSFVHSDTPLKLADYFNIRGVFKVGSIPDQPRRGGGIRLDTAVMGANHRA
FIEIVFQNREKIVQSYHLDGYSFWVVGMDRGTWSHASRREYNLVDAVSRSTTQVYPESWTAVYVALDN
VGMWNLRSEFWARQYLGQQLYLRVYSSVHSLRDEYLVPKNALLCGRASNMQRPIITP*
